# Directing Parthenogenetic Stem Cells Differentiate into Adipocytes for Engineering Injectable Adipose Tissue

**DOI:** 10.1155/2014/423635

**Published:** 2014-12-23

**Authors:** Wei Liu, Xingyuan Yang, Xingrong Yan, Jihong Cui, Wenguang Liu, Mei Sun, Yang Rao, Fulin Chen

**Affiliations:** ^1^Rege Lab of Tissue Engineering, Faculty of Life Science, Northwest University, 229 Taibai Road, Xi'an 710032, China; ^2^Department of Gynecology and Obstetrics, First Affiliated Hospital of Xi'an Jiaotong University, Xi'an 710061, China

## Abstract

The selection of appropriate seed cells is crucial for adipose tissue engineering. Here, we reported the stepwise induction of parthenogenetic embryonic stem cells (pESCs) to differentiate into adipogenic cells and its application in engineering injectable adipose tissue with Pluronic F-127. pESCs had pluripotent differentiation capacity and could form teratomas that include the three primary germ layers. Cells that migrated from the embryoid bodies (EBs) were selectively separated and expanded to obtain embryonic mesenchymal stem cells (eMSCs). The eMSCs exhibited similar cell surface marker expression profiles with bone morrow mesenchymal stem cells (BMSCs) and had multipotent differentiation capacity. Under the induction of dexamethasone, indomethacin, and insulin, eMSCs could differentiate into adipogenic cells with increased expression of adipose-specific genes and oil droplet depositions within the cytoplasm. To evaluate their suitability as seed cells for adipose tissue engineering, the CM-Dil labelled adipogenic cells derived from eMSCs were seeded into Pluronic F-127 hydrogel and injected subcutaneously into nude mice. Four weeks after injection, glistering and semitransparent constructs formed in the subcutaneous site. Histological observations demonstrated that new adipose tissue was successfully fabricated in the specimen by the labelled cells. The results of the current study indicated that pESCs have great potential in the fabrication of injectable adipose tissue.

## 1. Introduction

In plastic and reconstructive surgery, an increasing number of operations are involved in the repair of soft tissue defects resulting from deep burns, congenital diseases, and the removal of tumours ranging from small resections to mastectomy for breast cancer [1]. Autologous flap transplantations or commercial fillers, such as viscoelastic hylan and hyaluronic acid, are commonly used in soft tissue repair [2, 3]. Though having obtained a certain degree of success, these techniques have significant drawbacks, including donor-site morbidity and volume loss [4, 5].

Adipose tissue engineering is a growing field in regenerative medicine, and the strategy aims to fabricate functional adipose tissue with the combination of adipogenic cells and scaffold materials [6, 7]. Adult mesenchymal stem cells (MSCs) from the marrow and adipose tissue possess multipotent differentiation capacity and are commonly used as seed cells in adipose tissue engineering. However, these cells may lose their proliferative capacity and phenotype during* in vitro *expansion [8–10]. These drawbacks make it difficult to obtain adequate and functional cells for fabricating adipose tissue. Embryonic stem cells (ESCs) can differentiate into phenotypes of all three germ layers and are envisioned as a viable source to fabricate the desired tissue. Great efforts have been made to manipulate ESCs to differentiate into specific phenotypes and serve as an unlimited supply of seed cells. Detailed protocols to induce the differentiation of ESCs reliably and at high efficiency into adipogenic cells have been described [11, 12]. However, most current harvest techniques for ESCs require the destruction of embryos, which may lead to significant political and ethical concerns regarding their application [13].

Parthenogenesis refers to the embryonic development of eggs activated artificially without fertilization. Parthenogenetic embryonic stem cells (pESCs) can be obtained from the inner cell mass of parthenotes [14]. Because genetic defects affect normal placenta formation in human, parthenogenetic embryos are unable to grow into viable fetuses, and this characteristic allows the creation of pESCs avoiding the ethical hurdles associated with ESCs [15]. pESCs possess the typical characteristics of ESCs, including extensive self-renewal and differentiation* in vitro* and* in vivo* into cells of all three germ layers [16]. Human pESCs might be histocompatible with a significant portion of the population due to the presence of homozygous HLA genotypes [17, 18]. The common HLA haplotype-matched pESCs might reduce the risk of immunorejection after transplantation of their differentiated derivatives, offering significant advantages for the allogeneic applications of cell-based therapies over ESCs.

We hypothesized that pESCs can differentiate into functional adipogenic cells and be used for engineering adipose tissue. In the current experiment, we employed a stepwise approach to differentiate the pESCs into the cells of adipogenic lineage. The phenotype of the obtained cells was monitored by the expression of adipocyte specific genes and the accumulation of intracellular triglycerides. Then, the cells were seeded into Pluronic F-127, and the* in vivo* formation of adipose tissue through an injectable manner was investigated.

## 2. Materials and Methods

### 2.1. Cell Isolation and Culture

Mouse pESCs (derived from C57BL/6 mice, a kind gift from Professor Jinlian Hua, the Northwest A&F University, China) were cultured on a feeder layer of mouse embryonic fibroblasts inactivated with mitomycin C and expanded in ESGRO COMPLETE PLUS's medium (Millipore, Billerica, MA), in a 37°C/5% CO_2_ incubator. The cell morphology was observed by phase contrast microscope (TE2000-U, Nikon Inc.).

Bone morrow mesenchymal stem cells (BMSCs) were isolated and expanded from long bones of C57BL/6 mice. Briefly, long bones were excised into fragments and cultured in Dulbecco's Modified Eagle's Medium (DMEM, Sigma-Aldrich, St. Louis, MO, USA) containing 10% foetal bovine serum (Gibco, Grand Island, NY) for 3 d. The nonadherent cells and tissue debris were removed carefully and fresh medium was added. The cells were used for studies after 2 passages.

The pESCs were seeded on coverslips (Fisher Scientific) and fixed in 4% paraformaldehyde on ice. After being washed three times with phosphate buffered saline (PBS) and blocked with 10% normal donkey serum, the samples were incubated overnight with primary antibodies including OCT4, NANOG, and SSEA-1 (Santa Cruz Biotechnology, Dallas, TX) at 4°C. After three washes with PBS, the cell slides were incubated at room temperature with the secondary antibodies (Invitrogen, Carlsbad, CA) for 30 min. Images were taken with a laser confocal microscope (FV1000, Olympus Corporation, Tokyo). For negative controls, the primary antibody was omitted.

To evaluate if pESCs possess pluripotent differentiation capacity* in vivo*, the cells were trypsinized, and 1 × 10^6^ cells suspended in 50 *μ*L DMEM were injected subcutaneously into nude mice. After 4 weeks, the formed teratomas were harvested and fixed in 4% paraformaldehyde, dehydrated though graded ethanol, embedded in paraffin, sectioned (7 *μ*m sections), and stained with hematoxylin and eosin (H&E).

### 2.2. Derivation and Characteristics of Embryonic Mesenchymal Stem Cells (eMSCs) from pESCs

#### 2.2.1. EB Formation

The pESCs were trypsinized and resuspended in cell growth medium (CGM) (DMEM supplemented with 50 mg/mL L-proline (Sigma), 1% nonessential amino acids (Millipore), 1% 2-mercaptoethanol (Sigma), and 20% foetal bovine serum (Gibco)). The cells (1 × 10^5^) were transferred into ultralow attachment dishes (Fisher Scientific) and cultured to form EBs. The medium was changed every other day.

#### 2.2.2. Spontaneous Differentiation* In Vitro*

The pESCs were able to form EBs when cultured in suspension for 4-5 d as described above. Then the EBs were plated onto 0.1% gelatin-coated dishes to evaluate their developmental potential by monitoring the expression of specific markers. CGM was used for culturing EB outgrowths, and the outgrowths were allowed to differentiate for 10 d.

#### 2.2.3. Gene Expression Analysis by RT-PCR

To monitor the sequential expression of differentiation-related genes in the EBs and the outgrowths, RT-PCR studies were performed at 2, 4, 6, 8, and 10 d after EB plating. The total RNA was extracted from the cells using a RNA isolation reagent (Takara Bio Inc., Japan) according to the manufacturer's protocol. The extracted RNA was quantified using a GeneQuant pro (GE Healthcare, USA). The RevertAid First Strand cDNA Synthesis Kit (Thermo Fisher Scientific, Waltham, MA) was used to convert the RNA template into cDNA. The reaction was conducted in an Eppendorf Mastercycler (Eppendorf, Germany) with the following temperature profile: 65°C for 5 min, 25°C for 5 min, 50°C for 50 min, and 70°C for 15 min. The primer sequences and the fragment sizes were listed in Table 1. All the primers were obtained from Takara. The amplified products were separated on 1% (w/v) agarose gels and visualized with the assistance of ethidium bromide and ultraviolet light. *β*-actin was used as the reference gene.

#### 2.2.4. Derivation and Expansion of eMSCs

Eight days after EB plating, spindle-shaped cells could be observed in the outgrowths. The cells were then selectively separated by cell scrapers and subcultured in MesenCult MSC Basal Medium (StemCell, UK) at high density. The culture medium was changed every other day. The population of cells obtained was passaged after 14~21 d of culture. After 4~5 additional passages, the cells exhibited uniform spindle-shaped pattern.

#### 2.2.5. Characteristics of eMSCs

Cell proliferation was analyzed using cell proliferation reagent 4-[3-(4-iodophenyl)-2-(4-nitrophenyl)-2H-5-tetrazolio]-1,3-benzene disulfonate (WST-1, Roche) assay. For this procedure, eMSCs (p6) were trypsinized and 1000 cells in 100 *μ*L MSC Basal Medium were seeded in 96-well plates in quintuplicate. 10 *μ*L of WST-1 was added to each well and incubated for up to 7 d. The absorbance was measured every 24 h in a microplate reader (BioTek Synergy HT, Winooski, VT) at 450 nm. BMSCs of p3 acted as control.

The eMSCs (p6) were trypsinized, and 1 × 10^5^ cells were resuspended in 100 *μ*L of PBS. The cells were labelled with FITC-conjugated rabbit anti-mouse antigens CD13, CD14, CD29, CD44, CD45, CD90.2, CD105, CD133, and CD166 (BD Biosciences, Franklin Lakes, NJ). BMSCs of p3 were used as control. FITC-conjugated isotype-matching Igs were used to determine nonspecific staining. The cells were examined by a FACSCalibur cytometer, and the data were analysed with Cell Quest software (BD Biosciences).

The eMSCs (p6) were trypsinized and seeded on coverslips. After 4~5 d, cells were fixed in 4% paraformaldehyde for 30 min, incubated overnight at 4°C with primary antibodies for SFA-1, creatine kinase-M (CKM), Col1a1, Col2a1, integrin *β*1, vimentin (Santa Cruz Biotechnology), and heavy chain cardiac myosin (Abcam, Cambridge, United Kingdom). Immunocytochemistry was carried out as previously described.

### 2.3. Directed Differentiation of eMSCs

#### 2.3.1. Osteogenic and Chondrogenic Differentiation

For osteogenic differentiation, eMSCs (p6) and BMSC (p3) were cultured in osteogenic differentiation medium (CGM supplemented with 50 nM dexamethasone (Sigma), 10 mM beta-glycerophosphate (Sigma), and 50 *μ*M ascorbate-2-phosphate) for 21 d. The medium was changed every 3 d. RT-PCR was conducted to analyse gene expression profiles related to osteogenic lineage at 7, 14, and 21 d after induction. After 21 d, the cells were fixed in 4% paraformaldehyde and processed for Von Kossa and Alizarin Red S staining. For chondrogenic differentiation, eMSCs and BMSCs were cultured in chondrogenic medium (CGM supplemented with 50 *μ*M ascorbate-2-phosphate, 10 ng/mL TGF-*β*1 (R&D Systems, Minneapolis, MN), and 500 ng/mL IGF (R&D Systems)). The medium was changed every 3 d. RT-PCR was conducted to analyse gene expression profiles related to chondrogenic lineage at 7, 14, and 21 d after induction. After 21 d, the cells were processed for Safranin O and immunocytochemistry staining for Col2a1 and aggrecan. eMSCs cultured in normal MSC Basal Medium acted as control. The primers used were listed in Table 2.

#### 2.3.2. Adipogenic Differentiation

For adipogenic differentiation, eMSCs (p6) and BMSC (p3) were exposed to adipogenic induction medium (CGM supplemented with 1 *μ*M dexamethasone, 0.5 mM isobutylmethylxanthine, 200 *μ*M indomethacin, and 10 *μ*g/mL insulin (Sigma)) for 3, 6, 9, and 12 d. The medium was changed every 3 d. qRT-PCR was conducted to characterize the gene expression of key adipogenic markers (c/ebp, ppar-*γ*, ap2, and lpl). qRT-PCR was performed using the Bio-Rad real-time PCR system. The relative levels of gene expression were normalized to the *β*-actin gene using the comparative CT method according to the manufacturer's instructions. The primers used were listed in Table 3.

The cells were trypsinized and seeded on coverslips. After 3, 6, 9, and 12 d of induction, cells were fixed in 4% paraformaldehyde for 1 h, and immunocytochemistry was carried out as previously described. The primary antibodies included AP2, C/EBP, and LPL (Santa Cruz Biotechnology). The channel settings of the pinhole, detector gain, amplification offset, and gain were adjusted to provide an optimal balance of fluorescence intensity (channel 1: PMT voltage 294 V; channel 2: 443 V).

For Oil Red O staining, the cells were fixed in 10% formalin and washed with 60% isopropyl alcohol (Sigma). The cells were then incubated in 2% Oil Red O reagent (Sigma) for 15 min at room temperature. Excess stain was removed by washing with 60% isopropyl alcohol. The staining results were obtained by phase contrast microscopy. The proportion of adipogenic differentiation was determined by counting the Oil Red O-positive and Oil Red O-negative cells. Five fields of view were counted for each sample.

### 2.4. Implantation

#### 2.4.1. Animals

Six-week-old nude mice (male) were purchased from the Shanghai Experimental Animal Centre, Chinese Academy of Science. All the animal studies and protocols were processed following the guidelines of the Animal Holding Unit of Northwest University.

#### 2.4.2. Pluronic F-127 Preparation

Pluronic F-127 (Sigma) was slowly dissolved in DMEM at 4°C to prepare a 20% (w/v) solution. The solution was filter-sterilized through a 0.22 *μ*m pore size bottle-top filter and stored at 4°C before use.

#### 2.4.3. Injection and Observation

To identify adipocyte within the site of implantation* in vivo*, the adipogenically induced eMSCs (d6) were stained with CM-Dil, which is stable in subsequent experiments. Then the cells were seeded into ice-cold Pluronic F-127 to reach a cell concentration of 2 × 10^6^/mL. The solution was kept on ice to prevent premature gelation before injection. The gels (0.5 mL) were carefully transferred from the 15 mL centrifuge tubes into a 12-well plate (Corning), observed, and processed for Oil Red O staining.

The nude mice were anesthetized by intraperitoneal injection of 10% chloral hydrate (300 mg/kg), and the 0.5 mL cell-hydrogel composite was injected subcutaneously in the backs of the nude mice (*n* = 5). At 4 weeks after injection, the mice were sacrificed by 10% chloral hydrate, and the specimens were explanted for gross inspection and histological analysis.

#### 2.4.4. Transmission Electron Microscope (TEM)

For TEM, the cells were trypsinized, neutralized, centrifuged, rinsed, and routinely fixed in 1 mL of a fixative solution (2.5% glutaraldehyde and 2% formaldehyde) for 6 h at 4°C. The samples obtained from the* in vivo* experiments were fixed in the above fixative solution for 24 h at 4°C.

The cell and tissue samples were enrobed in 2% molten agarose, chilled at 48°C for 30 min, diced into 1 mm cubes for dehydration with graded ethanol, and embedded in EMbed812 epoxy resin (EMS, Hatfield, PA). Ultrathin sections were stained in 3% aqueous uranyl acetate in Sato triple lead stain prior to examination using an FEI CM12 Electron Microscope (FEI Co., Hillsboro, OR).

#### 2.4.5. Statistical Analysis

The data are expressed as the means ± S.E.M. Statistical significance was evaluated using SPSS for comparisons using unpaired Student's *t*-test (SPSS software, version 16.0). Values of *P* < 0.05 were considered significant.

## 3. Results 

### 3.1. Characterization of pESCs

**Figure 1:**
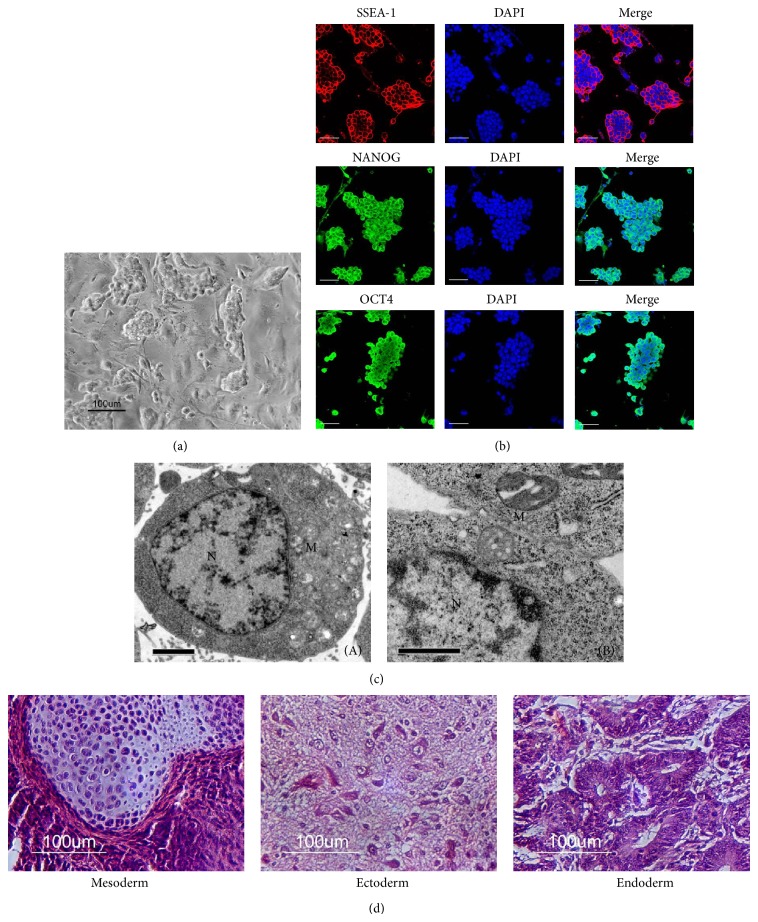
(a) Morphological observations of pESCs under phase contrast microscopy. (b) Immunocytochemistry staining of embryonic stem cell markers for pESCs (bars = 100 *μ*m). (c) Transmission electron microscopy shows the initial-stage of pESCs (p35), (A), (B): pESCs “N”: nucleus of cell; “M”: mitochondrion; bars = 2 *μ*m, 1 *μ*m. (d) H&E staining of teratoma sections from the pESCs. The staining revealed pESCs possessing the capacity to generate cartilage, neurocytes, and gastrointestinal tissue* in vivo*.

The pESCs maintained a typical saucer-shaped morphology, which was thickened at the rim and thinned out towards the centre with a high nuclear-cytoplasmic ratio and prominent nucleoli. Isolated pESCs could be expanded and form compact groups on MEF feeder cells (Figure 1(a)). The pESCs expressed high levels of SSEA-1, NANOG, and OCT4, which played a key role in maintaining the undifferentiated state of cells (Figure 1(b)). Transmission electron microscopy confirmed a high nuclear-cytoplasmic ratio, and the cytoplasm contained dense pools of glycogens and a few spherical or oval mitochondria (Figures 1(c)(A) and 1(c)(B)). To determine if the pESCs possessed* in vivo *pluripotent differentiation capacity, pESCs were injected into nude mice to test their ability to form teratomas. Tumour blocks could be observed on the backs of the animals 4 weeks after injection. Histological observations showed that the pESCs were able to form teratomas containing the three germ layers of gastrointestinal tissue (endoderm), cartilage (mesoderm), and neurocytes (ectoderm) (Figure 1(d)).

### 3.2. *In Vitro* Differentiation

**Figure 2:**
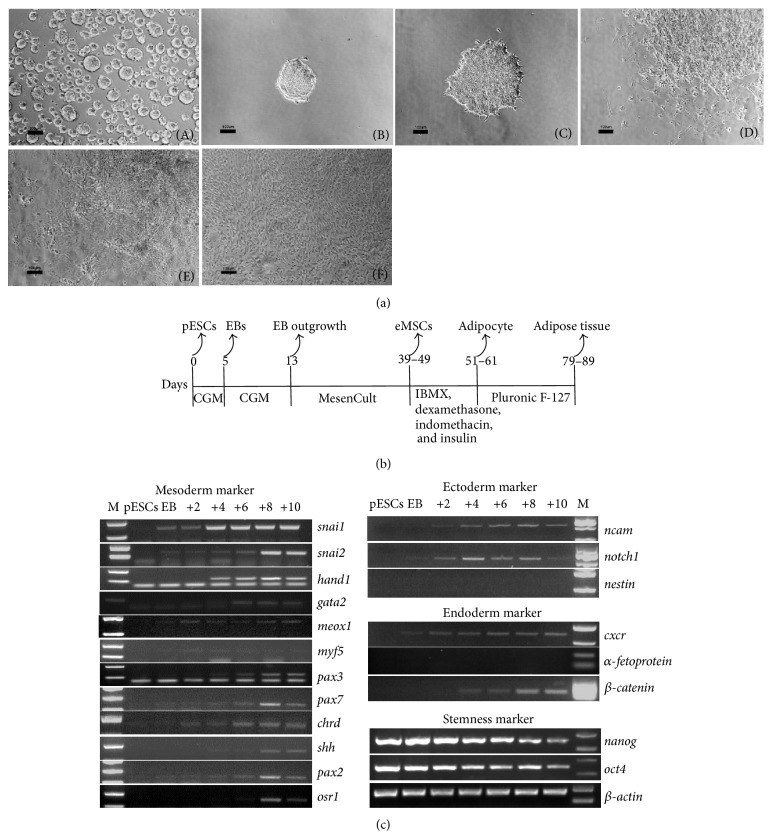
(a) Morphological appearance of pESC-derived EBs and outgrowths of EBs. (A) Micrograph of pESC-derived EBs. ((B)~(E)) Micrograph of outgrowths from 5 d EB plated on gelatin-coated plates for 2, 4, 6, 8, and 10 d. (F) Micrograph of eMSCs at passage 3 exhibiting spindle-shaped morphology (bars = 100 *μ*m). (b) Schematic outlines of pESCs differentiation and adipose tissue engineering flow chart. (c) Transcription of specific markers for mesoderm, endoderm, and ectoderm differentiation. RNA samples from undifferentiated pESCs, EBs (5 days in suspension culture) and (+): subsequent adhesion culture days were analysed by RT-PCR. Nanog and OCT4 are ESCs markers. *β*-actin served as an internal standard for RT-PCR. M. DL2000 DNA marker.

The pESCs could form EBs after 5 d of culture in ultralow attachment dishes. The EBs could attach to the gelatinized surface within 2 h and completely flattened within 2 d. The outgrowth cells displayed nonspecific irregular shapes (Figures 2(a)(A)–2(a)(C)) within the first 4 days after plating. At 6 d, several morphologically distinct populations of cells had appeared, forming outgrowths of spindle and brick-shaped cells (Figures 2(a)(D) and 2(a)(E)), and a typical undifferentiated morphology was observed in the densely packed colonies.

Gene expression profiling of the outgrowth cells from the pESCs-EB revealed the programmed expression pattern related to the differentiation toward ectodermal, mesodermal, and endodermal lineages. Kinetically, the Ncam1 and Notch transcription factors involved in the differentiation of the ectoderm began to be expressed on day 2, but nestin was not expressed. The endoderm-specific marker, cxcr4, began to be expressed on EB formation; *β*-catenin began to be expressed on day 4, respectively, and was sustained. Afp was not expressed. A gradual upregulation of genes involved in the epithelial-mesenchymal transition, snai1 and snai2, was observed. Other mesodermal subsets, including the lateral plate/extraembryonic mesoderm (hand1, gata2), axial mesoderm (chrd, shh), paraxial/myogenic mesoderm (meox1, myf5, pax3, and pax7), and intermediate (pax2, osr1) mesoderm genes, were upregulated. Pluripotent markers were expressed in the undifferentiated cells, and the expression decreased continuously after the beginning of differentiation in the study (Figure 2(b)). Collectively, these data indicated that the outgrowths of the EBs contained differentiated cells of the three germ layers, and among the above mesodermal markers, the snai2, hand1, pax3, pax7, shh, pax2 and osr1 transcription factors peaked on d 8 of differentiation.

### 3.3. Generation of eMSCs from EBs

According to gene expression levels for mesodermal commitment, the outgrowths of spindle-shaped cells were selectively isolated with a cell scraper 8 d after EB plating, and the cells were subsequently expanded in MSC Basal Medium to obtain eMSCs. After 4 to 5 passages, the population of cells obtained showed spindle-shaped morphology (Figure 2(a)(F)).

**Figure 3:**
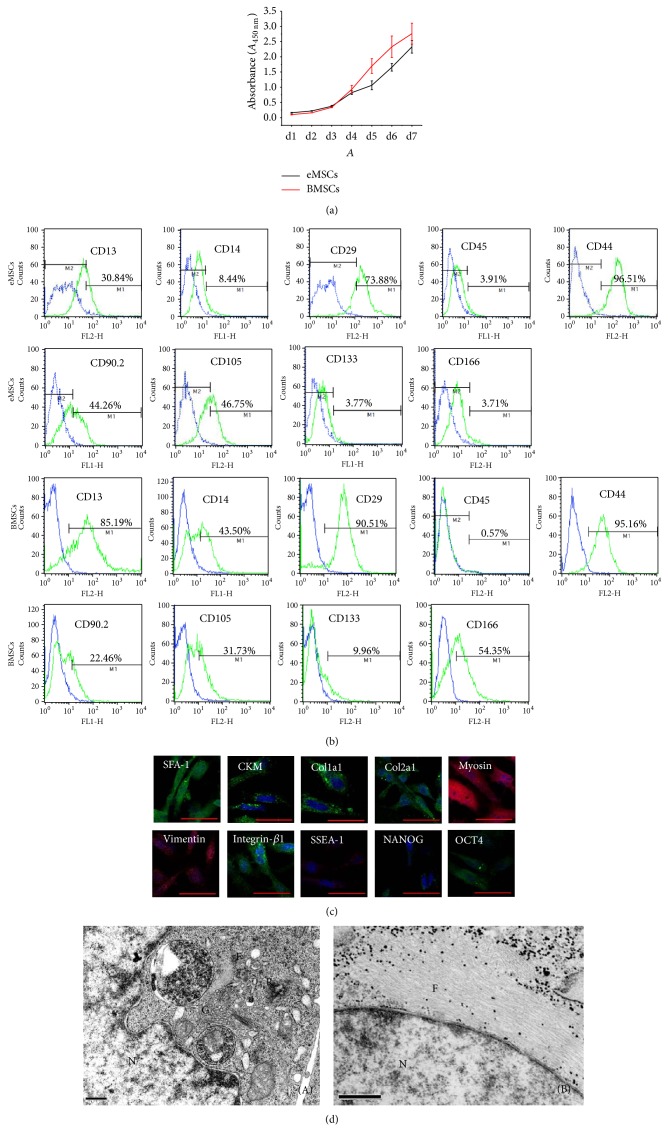
Characterization of eMSCs and BMSCs. (a) Growth curves of eMSCs and BMSCs. (b) Representative histograms of the cell surface marker expression on eMSCs (p6) and BMSCs (p3) analysed by flow cytometry, the labelled cells represented by the green line populations (M1), and relevant isotype-matched cells depicted by the blue line (M2). (c) Immunofluorescent staining for mesoderm and stemness markers. Antibody staining (green or red) with DAPI nuclear staining (blue) (bars = 50 *μ*m). (d) Transmission electron microscopy of eMSCs (p6). (A), (B): eMSCs bars = 0.5 *μ*m, “N”: nucleus of cell; “M”: mitochondrion; “F”: filaments fibres; “G”: Golgi apparatus; “R”: rough endoplasmic reticulum.

WST-1 assay showed that eMSCs had a significant faster proliferation rate than BMSCs (Figure 3(a)). Flow cytometry analyzed the surface marker of eMSCs. eMSCs were negative for CD45 (leukocyte common antigen), CD14 (lipopolysaccharide), and CD133 (aminopeptidase-N), indicating that they were not of hematopoietic origin. The adhesion molecule CD166 (activated leukocyte cell adhesion molecule) was not found to be expressed. The cells expressed CD29 (*β*1-integrin), CD13 (alanyl aminopeptidase), CD44 (hyaluronate receptor), CD90.2 (thymocyte differentiation antigen-1b), and CD105 (endoglin, SH2). The cell surface marker expression profiles indicated that the eMSCs were mesodermal cells. Surface marker expression patterns of eMSCs were similar to BMSCs (Figure 3(b)). Immunocytochemistry staining further confirmed that the expanded eMSCs exhibited mesodermal phenotypes (Figure 3(c)). Specifically, most of the eMSCs expressed SFA-1, CKM, Col1a1, Col2a1, ITGB1, myosin, and vimentin. The expression of Nanog, Oct4, and SSEA-1 was significantly decreased in eMSCs, compared with pESCs. Ultrastructural investigation showed that the cells contained exuberant nucleoplasm. The rough endoplasmic reticulum was extensive, and the mitochondria of the eMSCs contained distinct cristae. Filamentous fibres could be observed around the nucleus (Figures 3(d)(A) and 3(d)(B)).

### 3.4. Osteogenic Differentiation Analysis

**Figure 4:**
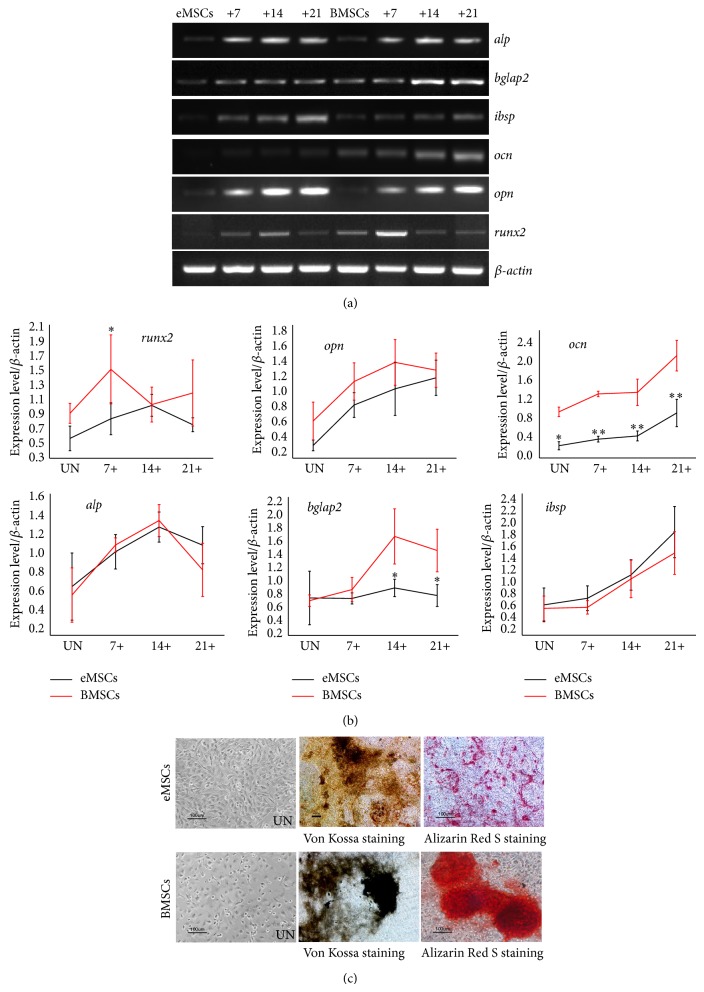
Osteogenic differentiation potential of eMSCs and BMSCs. (a) RT-PCR analysis of the gene expression profiles related to osteogenic differentiation. RNA samples from uninduced cells (UN) and induction cells (days +). (b) Semiquantitative analysis of gene expression levels by RT-PCR. RNA samples from uninduced cells (UN) and induction cells (days +). The relative expression of each gene was compared with the expression of *β*-actin in the same sample. Values are the means ± SD from 3 independent experiments. ^*^ *P* < 0.05, ^**^ *P* < 0.001. (c) Von Kossa and Alizarin Red S staining 21 d after osteogenic induction or uninduced cells (UN).

To confirm the multilineage differentiation capability of eMSCs, osteogenic differentiation was evaluated* in vitro *by using a standard protocol and compared with BMSCs. After being induced toward the osteogenic lineage, we compared the mRNA expression levels of osteogenic-specific markers at intervals of 7 d. RT-PCR results indicated that the expression of alkaline phosphatase (alp), bone gamma-carboxyglutamate protein 2 (bglap2), bone sialoprotein (ibsp), osteocalcin (ocn), secreted phosphoprotein 1 (opn), and runt related transcription factor 2 (runx2) was upregulated markedly after osteogenic induction (Figure 4(a)). Among these genes, ocn, runx2, and bglap2 were highly expressed in BMSCs compared to in eMSCs (Figure 4(b)). Strong staining for Von Kossa and Alizarin Red S 21 d after induction demonstrated that both cells had osteogenic potential, and BMSCs showed greater mineralization than eMSCs (Figure 4(c)).

### 3.5. Chondrogenic Differentiation Analysis

**Figure 5:**
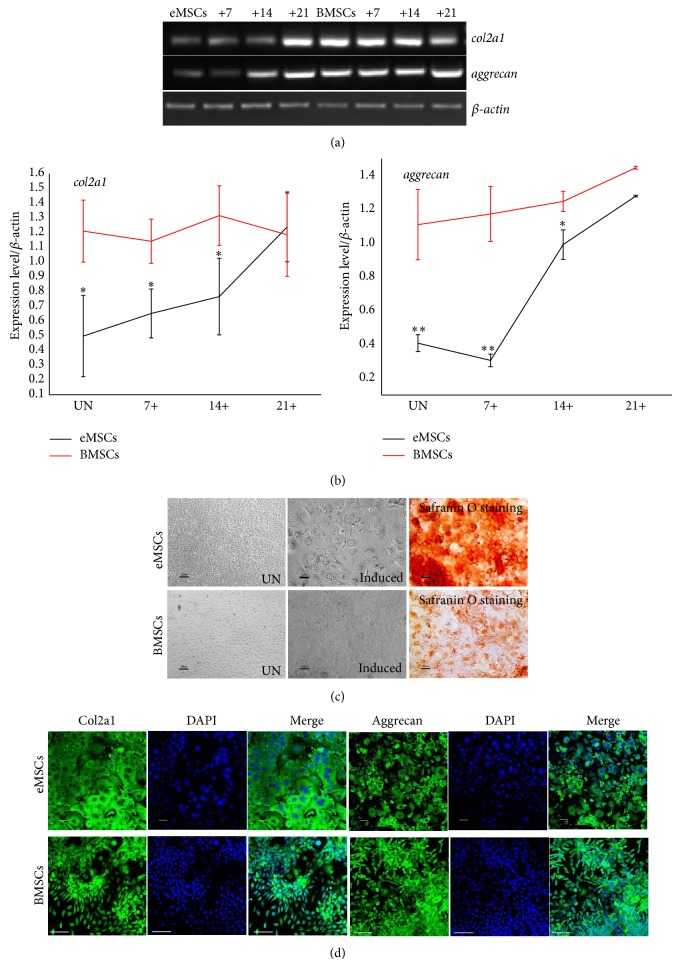
Chondrogenic differentiation potential of eMSCs and BMSCs. (a) RT-PCR analysis of the gene expression profiles related to chondrogenic differentiation. RNA samples from uninduced cells (UN) and induction cells (days +). (b) Semiquantitative analysis of gene expression levels by RT-PCR. RNA samples from uninduced cells (UN) and induction cells (days +). The relative expression of each gene was compared with the expression of *β*-actin in the same sample. Values are the means ± SD from 3 independent experiments. ^*^ *P* < 0.05, ^**^ *P* < 0.001. (c) Micrograph of cells 21 d after chondrogenic induction. Safranin O staining (red) for negatively charged proteoglycans 21 d after chondrogenic induction or uninduced cells (UN). Bars = 100 *μ*m, (d) immunofluorescent staining for aggrecan and Col2a1, antibody staining (green), with DAPI nuclear staining (blue).

RT-PCR, immunocytochemistry, and Safranin O staining verified the chondrogenic lineage differentiation of both cell types. The RT-PCR analysis showed that col2a1 and aggrecan were expressed in the induced and the uninduced eMSCs, and the expression increased following chondrogenic induction (Figure 5(a)). Chondrogenic differentiation-related genes, including col2a1 and aggrecan, were highly expressed in BMSCs compared with eMSCs (Figure 5(b)). Safranin O staining indicated that the cells produce basophilic ECM deposition. At 21 d after induction, both cells showed a typical spherical shape (Figure 5(c)) and well-defined COL2a1 and aggrecan staining in the cytoplasm and periphery of the cells (Figure 5(d)).

### 3.6. Adipogenic Differentiation Analysis

**Figure 6:**
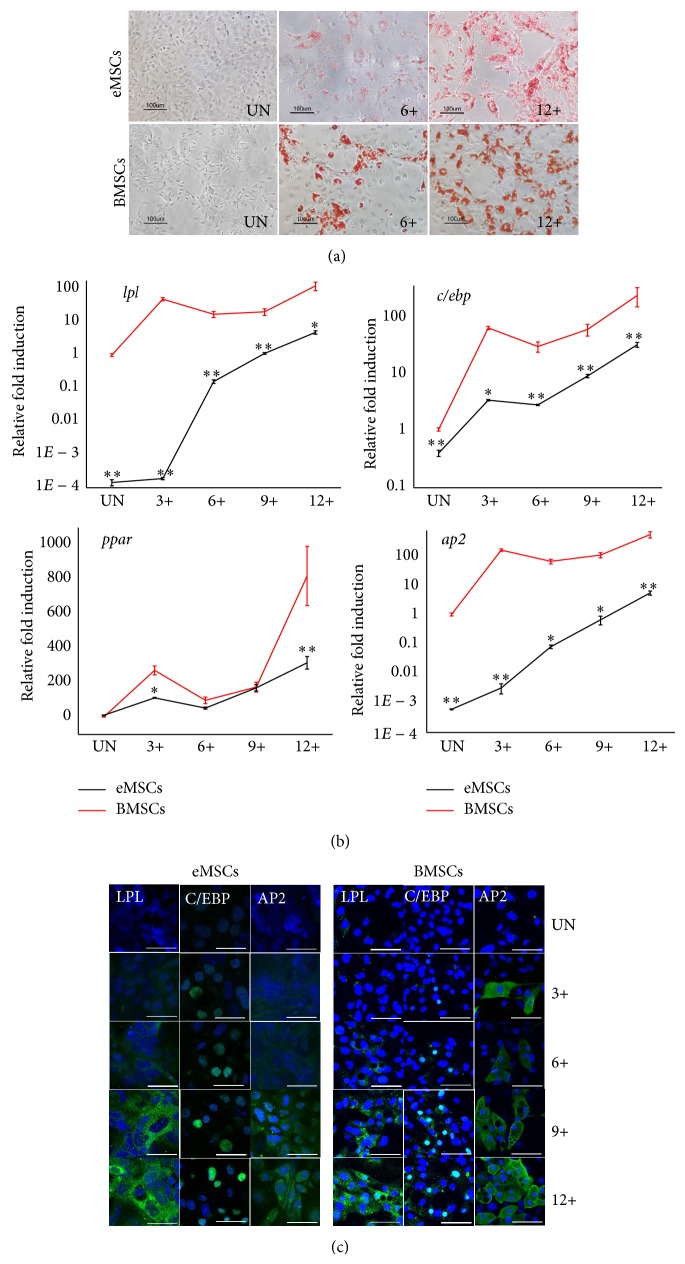
Adipogenic differentiation potential of eMSCs and BMSCs. (a) Lipid-containing adipocytes detected by Oil Red O staining after 12 d of culture in adipogenic medium. Samples from uninduced cells (UN) and induction cells (days +). (b) Gene expression profiles related to adipogenic differentiation in induced and uninduced (UN) cells. RNA samples from uninduced cells and induction cells (days +). Values are the means ± SD from 3 independent experiments. ^*^ *P* < 0.05, ^**^ *P* < 0.001. (c) Immunofluorescent staining for AP2, C/EBP, and LPL, antibody staining (green), with DAPI nuclear staining (blue). Samples from uninduced cells (UN) and induction cells (days +). Bars = 50 *μ*m.

To evaluate the adipogenic differentiation capacity of the eMSCs, both cells were subjected to a standard adipogenic induction culture. Oil Red O staining revealed that small lipid droplets formed in cytoplasm 6 d after induction. On day 12, the size of the lipid droplets increased significantly and occupied most of the cytoplasm. No Oil Red O-positive cells were observed in the uninduced specimens (Figure 6(a)). Cell counting result showed that 51% ± 6.8% and 39% ± 5.3% Oil Red O-positive cells were observed in BMSCs and eMSCs after 12 d of adipogenic induction (*P* < 0.05).

qRT-PCR was performed to evaluate the expression profile of specific gene markers of adipogenic cells, including lpl (lipoprotein lipase), c/ebp*α* (CCAAT/enhancer binding protein), ppar-*γ* (peroxisome proliferator activated receptor *γ*), and ap2 (adipocyte fatty acid binding protein). The expression levels of the BMSCs were used as reference values (set at 1) for the calculation of the *n*-fold change during the induction steps. After adipogenic induction, the eMSCs showed a significantly increased expression pattern for the four genes examined. Significant peaks in the expression of c/ebp*α* (33.91 ± 3.34-fold) were detected at day 9, of ppar-*γ* at day 9 (315.55 ± 36.47-fold), of lpl at day 12 (4.93 ± 0.57-fold), and of ap2 at day 12 (6.14 ± 0.99-fold) (Figure 6(b)). Expression of adipogenic-related genes, including c/ebp*α*, lpl, and ap2, was higher in BMSCs following induction than in eMSCs (Figure 6(b)).

Immunocytochemistry studies were performed to further demonstrate if the changes in mRNA expression resulted in elevated protein levels. As shown in Figure 6(c), the C/EBP*α* proteins were detectable in both cells as early as 3 d after induction, and then the expression increased steadily to 12 d after induction. The LPL antibodies were detectable in both cells at 6 d after induction. The aP2 antibodies were detectable at 3 d after induction in BMSCs, while eMSCs had a delayed signal and could be observed 6 d after induction.

### 3.7. Evaluation of Adipose Tissue Engineering* In Vivo*

**Figure 7:**
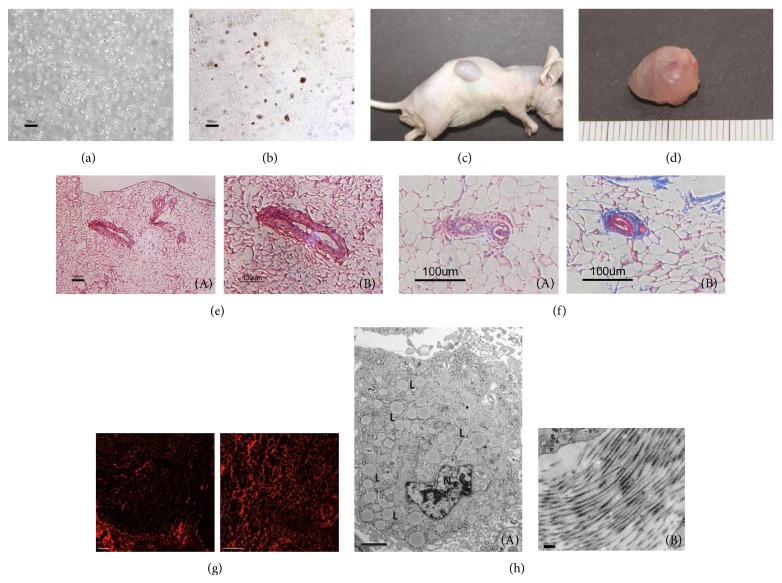
(a) Homogeneous cell encapsulation and high viability of embedded cells in the Pluronic F-127. (b) Oil Red O staining showed lipids in Pluronic F-127. (c) Subcutaneous injection of adipogenically induced eMSCs-hydrogels implants in nude mice after 4 weeks. (d) Gross view of the newly formed adipose tissue. (e), (f) H&E ((e)(A), (e)(B)) and Masson's trichrome ((f)(A), (f)(B)) staining of adipose tissue sections showed that mature adipocytes and functional blood vessels are observed in the specimen, bars = 100 *μ*m. (g) The CM-Dil labeled cells in tissue sections from the newly formed tissue. Bars = 100 *μ*m. (h) Transmission electron microscopy shows the implants (A) and extracellular matrix (B), “N”: nucleus of cell; “L”: lipid droplet, bars = 2 *μ*m, 200 nm.

Phase contrast microscopy observations showed that the adipogenically induced eMSCs were homogeneously distributed in the hydrogels and displayed a rounded morphology (Figure 7(a)). Lipid droplets could be noticed within the cells after Oil Red O staining (Figure 7(b)).

Four weeks after the injection, all the animals survived and no inflammation or extrusion occurred throughout the experiments. The newly formed tissue grafts were visible on the back of the animal (Figure 7(c)). There was a slight reduction in the volume of the grafts during the experiments. Gross inspection showed that the newly formed tissue was glistering and was semitransparent (Figure 7(d)). Histological observations revealed that the engineered adipose tissue recapitulated the major structure and the components of normal adipose tissue. A typical ring volume consisting of mature adipocytes and functional blood vessels could be observed in the specimen. Most of the hydrogel was absorbed, and no inflammatory cell infiltration or teratoma formation could be noticed (Figures 7(e) and 7(f)). The red fluorescent signal of CM-Dil labelled adipocytes was distributed in the newly formed tissue (Figure 7(g)).

The results of the TEM showed that the adipocytes in the newly formed tissue were mature and had a well-developed nucleolus, a cytoplasm rich in ribosomes and polyribosomes, glycogen clusters, small lipid droplets, and large, dense mitochondria with variable amounts of cristae. The ECM of collagen fibres could be observed in the specimen (Figure 7(h)).

## 4. Discussion

Development of a clinically translatable method to engineer adipose tissue for soft tissue reconstruction requires the coordination of several key aspects, including the selection of the cell source, the scaffold material, and the means of graft delivery [8, 19, 20]. The choice of an appropriate cell source is crucial for the strategy of tissue engineering. One key characteristic for the ideal cell type for adipose tissue engineering is that the source is available in sufficient quantities either by direct harvest or by* in vitro* expansion without losing the adipogenic phenotype [21]. ESCs are pluripotent and capable of producing a large range of specific phenotypes and have been considered as potential candidates for an unlimited source of cells for tissue engineering [22–25]. However, harvesting of ESCs may destroy viable embryos and lead to significant political and ethical concerns regarding their application. pESCs overcome the political and ethical hurdles associated with ESCs because pESCs originate from unfertilized oocytes. Importantly, pESCs have similar characteristics to ESCs, including the capacity of extensive self-renewal and differentiation into cells of all three germ layers [26]. As demonstrated in the current experiments, pESCs had the morphological characteristics of ESCs, exhibit the typical stemness markers, including Nanog, OCT4, and SSEA-1, and could form teratomas including three germ layers (Figure 1).

In the current study, we employed a stepwise strategy for directing pESCs to differentiate into adipogenic cells. We obtained EBs from the pESCs by suspension culture, and the gene expression patterns were investigated to monitor cell differentiation. Consistent with the* in vivo* teratoma formation capacity, the cells in the EB could be differentiated into three germ lines* in vitro*, as demonstrated by the expression of ectoderm (ncam1 and notch), mesoderm (snai1 and snai2), and endoderm (*β*-catenin and cxcr4) genes (Figure 2). Interestingly, no substantial expression of afp genes was also observed in the experiment, which was consistent with previous study [27]. Whether this characteristic is dependent on the species variation or parthenogenesis needs to be further investigated with other parthenogenetic ESC lines.

The EBs were plated on cell culture dishes to let cells outgrow. Outgrowths from EB exhibited different morphologies (Figure 2(a)(E)), and the spindle-shaped cell could be selectively collected and expanded to obtain eMSCs. Flow cytometry and immunocytochemistry staining confirmed that eMSCs expressed MSC markers (Figure 3(b)) and could be further differentiated into osteoblasts, chondrocytes, and adipocytes (Figures 4, 5, and 6).

It has been demonstrated that adipogenic differentiation could be induced by a cocktail consisting of IBMX, dexamethasone, indomethacin, and insulin. After exposure to the cocktail, the transcription factor CCATT enhancer binding protein *β* (C/EBP*β*) is activated. “Activated” C/EBP*β* triggers transcription of peroxisome proliferator activated receptor *γ* (PPAR*γ*) and C/EBP*α*, which in turn coordinately activate genes encoding the adipogenic phenotype [28, 29]. We found that lpl, ppar-*γ*, and c/ebp*α* were almost undetectable in the eMSCs, but their expression steadily increased from day 3 of induction and reached maximal levels at day 9. This was followed by the expression of mature adipocyte markers ap2 (adipocyte fatty acid binding protein), and the signals were strongly expressed on day 12 (Figure 6(b)). Immunocytochemistry and Oil Red O staining results further confirmed the above results (Figures 6(a)–6(c)) and demonstrated the functionality of obtained adipogenic cells.

Our results showed that eMSCs showed relatively lower osteogenic, chondrogenic, and adipogenic differentiation capacity compared with BMSCs. 12 d of adipogenic induction, Oil Red O-positive cells in eMSCs and BMSCs were 39.8% ± 5.3% and 51.2% ± 6.8%, respectively. However, a fundamental challenge in adipose tissue engineering is the provision of sufficiently large cell populations with appropriate adipogenic phenotype. eMSCs possessed higher proliferation rate than BMSCs (Figure 3(a)). Furthermore, it has been demonstrated that BMSCs may lose their multipotentiality during* in vitro *expansion [30].

Taken together, eMSCs may be more suitable for the engineering of adipose tissue of large volume. Screening and seeking for pESCs line with higher adipogenic differentiation capacity and development of optimised adipogenic induction protocol should be encouraged for the adipose tissue engineering with pESCs.

Scaffold and the delivery manner are two other key aspects for adipose tissue engineering. The biochemical and biomechanical properties of the scaffold have recently been shown to play a role in stimulating stem cell differentiation towards several lineages [31]. Currently, natural or synthetic materials with open porous structures are commonly used, and the fabricated grafts are implanted by open surgery [32, 33]. Culturing adipogenic stem cells on gels that mimicked the native stiffness of adipose tissue (2 kPa) significantly upregulated the adipogenic markers in the absence of exogenous adipogenic growth factors and small molecules. As the substrate stiffness increased, the cells became more spread out, lost their rounded morphology, and failed to upregulate adipogenic markers [34]. Pluronic F-127 is a synthetic hydrogel that is nontoxic, biocompatible, bioabsorbable, and FDA approved for use in humans and has been widely used in drug delivery and controlled release applications [35]. Differentiating BMSCs with small lipid droplets were observed even in the absence of adipogenic stimuli at day 14 in Pluronic F-127 [36]. This suggested that the 3D environment has some adipogenic differentiation signalling potential that is independent of hormonal induction, which emphasised the permissibility of Pluronic F-127 for adipogenesis. In addition, injectable materials allow for minimally invasive delivery, which reduces the surgical complications of implantation and prevents excessive scarring.

It is suggested that the adipose tissue engineering approaches should coordinate the processes of adipogenesis and neovascularisation [5, 37–39]. The* in vivo* injection experiment showed that mature and vascularised adipose tissue was successfully fabricated in the subcutaneous site 4 weeks after injection, and labelling of CM-Dil further confirmed that the newly formed tissue derived from implanted cells (Figure 7). Conclusively, the results of the current study support our hypothesis that pESCs could be directed to differentiate into functional adipogenic cells and be applied for adipose tissue engineering.

## 5. Conclusion

This study successfully directed pESCs to differentiate into adipogenic cells that expressed master genes for adipogenesis, including LPL, PPAR*γ*, AP2, and C/EBP*α*, and the cells could accumulate lipids in the cytoplasm. The induced adipogenic cells could be used to fabricate injectable adipose tissue in combination with Pluronic F-127 hydrogel, as demonstrated by gross inspection and histological observations. This novel approach might have the advantage of producing functional and ready-for-use allogeneic soft tissues for reconstructive and cosmetic surgery. The next issue we will address is to systematically evaluate the long-term histocompatibility of tissue-engineered adipose in immunocompetent animal models.

## Figures and Tables

**Table 1:** The primers sequence for RT-PCR.

Gene	Primers	Product
Snai1	GACCTGTGGAAAGGCCTTCTCTAGG	170 bp
	CCTGGCACTGGTATCTCTTCACATC	
Snai2	GGGAGCATACAGCCCTATTACTG	146 bp
	CCTTGGATGAAGTGTCAGAGGAA	
Hand1	GCTACGCACATCATCACCATCATC	125 bp
	CAGCAGCCAGCTCTGGAAGTAAG	
GATA2	GCCAAAAGAGAGACTGGAGGAAGGG	82 bp
	ACACCTCCCACCTTTTAGTCACTCTG	
Meox1	CAGTCAAAATGTTCAGCATGGTAG	195 bp
	AGAGGAAAATGTTGAATGGAAACTTTA	
Myf5	CCTGAAGAAGGTCAACCAAGCTTTC	158 bp
	GGCTGTAATAGTTCTCCACCTGTTC	
PAX3	CTCTGAACCTGATTTACCGCTGAA	124 bp
	CCTGGTGTAAATGTCTGGGTAGTG	
Pax7	CCTCAGGTCATGAGCATCCTTAGC	192 bp
	GTAGGTGGGTGGGCAGTAAGACTG	
Chrd	GGTGCAAGTGGTAGGTACAGGTAG	182 bp
	GCTCGTTCTGTAGCAGCATATGAG	
SHH	CCCAATTACAACCCCGACATCATATTTAAG	196 bp
	CCTCATAGTGTAGAGACTCCTCTGAATG	
PAX2	CTGTCCCTAATGGAGACTCCCAGA	162 bp
	CCTGTTCTGATTTGATGTGCTCTGATG	
OSR1	GCCACTTCACTAAGTCCTATAACCTAC	169 bp
	TTCCCACACTCTTGACACTTGAAA	
*α*-fetoprotein	CCCTCATCCTCCTGCTACATTTC	145 bp
	GGAACAAACTGGGTAAAGGTGATG	
*β*-catenin	GCAACCCTGAGGAAGAAG	501 bp
	TCCCAGCAGTACAACGAG	
CXCR4	CCACGGAGTCAGAATCCTCCAGTT	572 bp
	CCAGCATTTCTACCACCATTTCAGG	
NCAM1	CGACTCCTCTACCCTCACCATCT	559 bp
	CTCGTCATCTTCCTCCTCGTTCT	
NESTIN	GTTACCAAAGCCTCTTAGAAATGACC	577 bp
	CAGATGCAACTCTGCCTTATCCTC	
NOTCH1	AATGTGGATGCTGCTGTTGTGCT	569 bp
	ATGGATGGAGACTGCTGGAATGG	
NANOG	GCACTCAAGGACAGGTTTCAG	169 bp
	GACCATTGCTAGTCTTCAACCAC	
OCT3/4	GTGTGAGGTGGAGTCTGGAG	182 bp
	AGCCTCATACTCTTCTCGTTGG	
*β*-actin	GAGACAACATTGGCATGGCTTTG	190 bp
	CCTCAGCCACATTTGTAGAACTTTG	

**Table 2:** The primers sequence for RT-PCR.

Gene	Primers	Product
ALP	TCACGGCCATCCTATATGGTAAC	98 bp
	CTGGTAGTTGTTGTGAGCGTAATC	
OPN	CACACAGACTTGAGCATTCCAAA	77 bp
	GGAACTTGCTTGACTATCGATCAC	
RUNX2	CCAGTCTTACCCCTCCTATCTGA	138 bp
	GTGGCAGTGTCATCATCTGAAATAC	
OCN	CCATCTTTCTGCTCACTCTGCTG	136 bp
	CGGAGTCTGTTCACTACCTTATTGC	
IBSP	GAGACGGCGATAGTTCCGAAGAG	132 bp
	CCGAGAGTGTGGAAAGTGTGGAG	
BGLAP2	CAAGCAGGAGGGCAATAAGGTAG	133 bp
	CTGGTCTGATAGCTCGTCACAAG	
COL2a1	CCAGAACATCACCTACCACTGTAA	111 bp
	GCCCTCATCTCTACATCATTGGA	
AGGRECAN	CCATGTGTGGGTGACAAAGACAG	92 bp
	TCCACGTAGCAGTAGACATCATAGG	

**Table 3:** The primers sequence for qRT-PCR.

Gene	Primers	Product
C/EBP	CCGATGAGCAGTCACCTCCAGAG	169 bp
	GGTCGATGTAGGCGCTGATGTCTA	
PPAR-*γ*	CACCAACTTCGGAATCAGCTCTG	83 bp
	CTGTGGTAAAGGGCTTGATGTCAA	
AP2	ACAGGAAGGTGAAGAGCATCATAA	143 bp
	GGAAGTCACGCCTTTCATAACAC	
LPL	GCTGTAACAATCTGGGCTATGAGA	78 bp
	GAGAGCGAGTCTTCAGGTACATC	
